# Nectar traits of *Echium vulgare* in urban habitats are associated with local temperature, floral traits, and surrounding water bodies

**DOI:** 10.1186/s12862-026-02574-0

**Published:** 2026-08-14

**Authors:** Barbara Płaskonka, Marcin Zych, Katarzyna Roguz

**Affiliations:** Botanic Garden, Faculty of Biology, University of Warsaw, Aleje Ujazdowskie 4, Warszawa, 00-478 Poland

**Keywords:** Urban Heat Island, Floral rewards, Physiological buffering, Water, Plant-pollinator interactions, Ruderal vegetation

## Abstract

**Background:**

Urbanization exposes plants to multiple environmental stressors, including landscape-level changes in land-cover composition (domination of impervious surfaces, reduced proportion of green areas and water bodies), elevated temperatures associated with the Urban Heat Island effect, and altered water availability. These factors can alter nectar production and quality, with potential consequences for plant–pollinator interactions and the stability of ecosystem services in urban environments. However, the relative importance of different components of urban environmental variation — including landscape composition, temperature, and conditions experienced at the plant level — remains poorly understood.

**Results:**

We quantified nectar volume and sugar concentration in 800 flowers and analyzed their relationships with floral morphology, flower position within inflorescences, air temperature, and the proportion of impervious surfaces, green areas, and water bodies (within radii: 150, 600, and 1000 m). Coarse urbanization metrics did not explain variation in nectar traits, highlighting limited predictive power of land-cover structure for floral reward dynamics in urban ecosystems. Neither impervious surfaces nor green area cover significantly influenced nectar volume or sugar concentration at any spatial scale. Instead, nectar production was better explained by local thermal conditions, proportion of water bodies cover, and floral morphology. Nectar volume increased with air temperature and with the proportion of water bodies within 600 m, while sugar concentration showed a marginal positive association with water bodies at the 1000 m scale and tended to decrease under warmer conditions. Larger flowers produced greater nectar rewards, and flower position within the inflorescence significantly affected both nectar volume and concentration.

**Conclusions:**

Our results suggest that nectar production in urban E. vulgare populations is influenced more strongly by local thermal conditions, proportion of water bodies cover, and floral traits than by broad land-cover composition, indicating that fine-scale environmental heterogeneity and specific landscape elements may play a key role in maintaining floral rewards for pollinators in urban ecosystems.

## Background

Pollination represents a fundamental ecosystem service, vital to the maintenance of both wild plant communities [42] and agricultural productivity [27]. Without pollinators, many plants could not set seed and reproduce. Conversely, without plants providing pollen, nectar and other rewards, many animal populations would decline, with consequent knock-on effects for other related species [26, 39]. Human reliance on animal-pollinated crops demonstrates the value of the ecosystem services provided by pollinators: 75% of the world’s leading food crops show increased fruit or seed set with animal pollination [27]. Similarly, the integrity of natural ecosystems is profoundly linked to these interactions, as nearly 90% of all wild flowering plant species globally depend on animal vectors for sexual reproduction [39], making pollinators indispensable for the persistence of biodiversity and the stability of terrestrial habitats.

Despite its critical importance, the stability of pollination is increasingly threatened by global environmental changes [42]. As global urbanization is one of the causes for this situation and, at the same time, an irreversible trend, with more than 60% of the world’s population projected to live in cities by 2030, the focus of conservation biology must expand (United Nations 2018/2019). While protecting wild species in their natural habitats remains a priority, it is equally vital to safeguard ecosystem services within highly transformed environments [2]. Recent evidence suggests that this is indeed possible; urban landscapes, despite their fragmentation, can paradoxically serve as biodiversity refugia [3, 5]. The capacity of cities to provide stable and safe pollination habitat depends on how this environment is shaped [20, 45] and how plants and their associated visitors respond to anthropogenic pressures [1, 40, 41]. Plants in cities are subject to intense environmental filters, such as elevated temperatures, wind turbulence, or changes in the duration of growing season, which often lead to significant alterations in their functional traits [1]. These changes, such as shifts in flowering phenology [35] or modified reproductive allocation [12, 17], are direct responses to the Urban Heat Island (UHI) effect and soil sealing. While attention has been paid to shifts in flowering phenology and vegetative morphological traits (e.g., Bucharova et al. [11], Irwin [23]), understanding how urban stressors influence ‘hidden’ traits, such as nectar properties, is essential for maintaining the functioning of urban ecosystems. Nectar is the primary floral reward globally, consumed by a broad spectrum of visitors [53]. Its vital role in plant reproduction is underscored by the fact that approximately 74% of biotically-pollinated angiosperms rely on nectar to attract animal vectors [4].

The urban environment exerts a unique set of physicochemical pressures on plant physiology, primarily through the synergism of the UHI effect and extensive soil sealing (impervious surfaces). Elevated ambient temperatures in cities [38] can directly accelerate metabolic rates but also pose a significant risk of thermal stress to floral tissues. For nectar-producing plants, this thermal regime creates a challenging trade-off: while higher temperatures can initially stimulate nectar secretion by increasing metabolic activity, they simultaneously enhance evaporative water loss from the corolla [15]. Soil sealing further modifies local hydrological dynamics: while it can increase stormwater runoff into unsealed patches — causing transient periods of elevated soil moisture — it simultaneously restricts groundwater recharge and lowers atmospheric humidity, often leading to subsequent severe aridity [52]. Under such fluctuating microclimatic conditions, plants often experience stress related to the need of maintaining nectar volume attractive to pollinators versus conserving water to ensure survival [15]. While extreme heat and water scarcity can render these rewards energetically unprofitable [17], empirical data on how the interplay between the UHI, hydrological and landscape features shapes nectar in resilient, synanthropic flora remains scarce.

To investigate these dynamics, we selected *Echium vulgare* L. (Boraginaceae) as a model species. *Echium vulgare* is a biennial, synanthropic “urban winner” known for its exceptional resilience to anthropogenic disturbances [28] and important role in shaping urban nectar resources [15], which makes it an ideal subject for studying variation in nectar traits in urban areas.

The primary objective of this study was to evaluate the role of local thermal conditions, floral morphology and landscape-scale predictors in shaping the nectar traits of a resilient urban specialist. We specifically tested the following hypotheses: (1) At the landscape scale, nectar traits in *E. vulgare* will show limited variation across broad urbanization gradients. (2) Proximity to green areas, blue infrastructure and local temperature shape nectar volume and concentration.

Landscape-scale variables were defined as spatial land-cover characteristics (the proportions of impervious surfaces, green areas and water bodies) quantified within three circular buffers (radii: 150 m, 600 m, and 1000 m) centered on each population.

## Methods

**Fig. 1:**
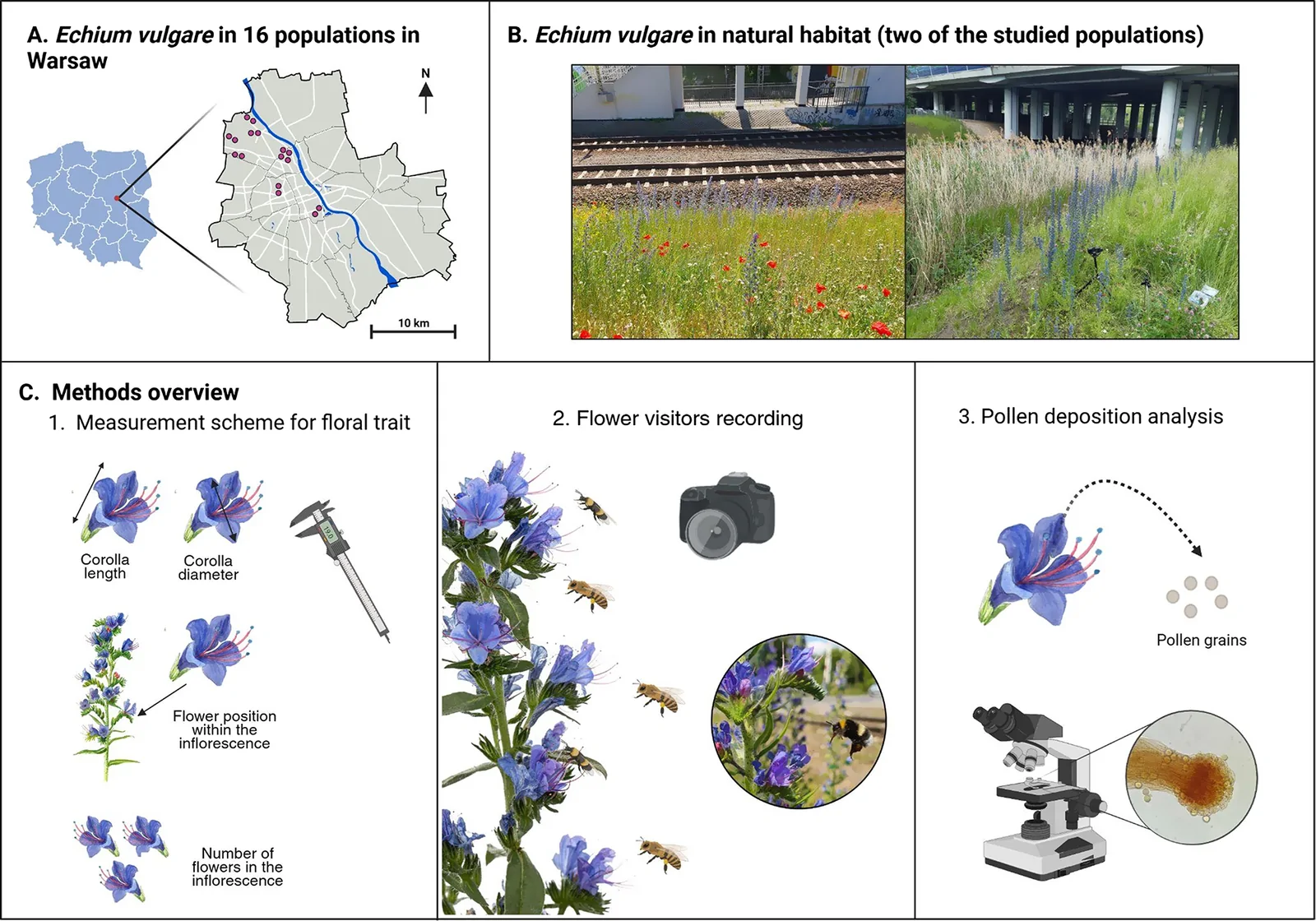
Overview of the study design and sampling protocol used to assess the effects of urban environmental conditions and floral morphology on nectar traits of *Echium vulgare* in Warsaw. Created with BioRender.com

To evaluate the impact of urbanization on floral traits, nectar samples of *E. vulgare* were collected and analyzed over two consecutive flowering seasons. Fieldwork was conducted in Warsaw, Poland, at 16 distinct study sites. Sampling took place between May 30 and June 8, 2023, and from May 17 to June 4, 2024 (Fig. 1).

### Study species

*Echium vulgare* L. is a self-compatible monocarpic perennial [44] flowering from June to September [31]. The species is native to most of Europe and is widespread in the flora of Poland, where it commonly occurs in ruderal phytocoenoses, dry hills, in rocky debris and stone embankments [46, 47]. Its high tolerance for nutrient-poor and arid conditions makes it a characteristic component of urban spontaneous flora, where it acts as a resilient floral resource for pollinators amidst fragmented habitats. Individual plant may produce 1–20 inflorescences, each composed of up to 20 cymes. While an individual may produce hundreds of flowers, floral display is regulated by staggered blooming, with typically only a few flowers open simultaneously per cyme [29, 44]. Individual flowers (15–20 mm in length) have a pubescent, tubular corolla containing easily accessible nectar (0.05 to 0.6 µl per flower). Nectar secretion patterns are dynamic, peaking in the morning and evening and decreasing with floral senescence [14, 31]. Consequently, *E. vulgare* supports a broad spectrum of anthophilous insects: bumblebees [14, 32], honeybees [14] and solitary bees (Strickler, 1979), but other insect visitors have also been recorded [14], a total of approximately 200 insect species [19].

### Nectar sampling

To assess the effects of urban conditions on nectar traits, samples were collected at approximately 7:30 h. To ensure the measurement of nectar production and to prevent depletion by floral visitors, five inflorescences per site were bagged at the bud stage using fine-mesh exclusion nets. From each isolated inflorescence, five flowers were sampled, totaling 800 flowers across the 16 study sites within 2 consecutive seasons (5 flowers x 5 inflorescences x 16 study sites x 2 seasons). To ensure independence, flowers were sampled from inflorescences from five distinct individual plants whenever possible. Only in rare instances of extremely small number of flowering individuals, were flowers taken from inflorescences (not more than two) from a single large individual. For each sampled flower, corolla length and diameter were measured to the nearest 0.1 mm using a digital caliper. To account for intra-inflorescence variation, the relative position of each flower was recorded, calculated as the sequential rank of the flower from the base divided by the total number of cymes on the inflorescence. Nectar was extracted using glass microcapillaries (10 µl). The length of the nectar column in the capillary was measured with a digital caliper in mm and subsequently converted to volume (µl). Nectar samples were placed in 1.5 ml Eppendorf tubes and immediately stored at -20 °C for further analysis. Sampling was strictly conducted during favorable weather conditions. In the event of precipitation, which occurred on two sampling dates, operations were suspended to avoid nectar dilution [16] and resumed only on the following suitable sunny day. In the laboratory, nectar sugar concentration (%, sucrose equivalents) was determined using an Abbe refractometer (RL-4).

### Habitat characteristics

The study encompassed 16 populations studied in two consecutive years. Population size varied, with the number of inflorescences ranging from 15 to 200 inflorescences in 2023, and from 15 to 300 in 2024.

If a population was not accessible in a second year due to mowing, absence of flowering individuals, or anthropogenic disturbance, it was replaced by a nearby population located within 400 m from the original site. In such cases, the replacement population was assigned a new unique ID to distinguish it from the original population in subsequent analyses.

To assess the effects of landscape on each population, the proportion of impervious surfaces, green areas and water bodies within three circular buffers (radii: 150, 600, and 1000 m) centered on each population were calculated. These metrics were calculated as a ratio of the specific land-cover type to the total area of all other categories (e.g., for the proportion of green areas: green areas / (green areas + impervious areas + area of water bodies)). Data related to urbanization were obtained from freely accessible geographic information system (GIS) datasets, specifically the Database of Topographic Objects (BDOT10k). This database provides information with a positional accuracy corresponding to topographic maps at a 1:10,000 scale (https://cyfryzacja.mazovia.pl/darmowe-dane.html). Spatial parameters were analyzed using QGIS version 3.4.11 ‘Madeira’ (QGIS, 2019).

Local environmental stressors were characterized using thermal data. Air temperature at 7:00 (2 m above ground level) was extracted from the ERA5-Land reanalysis dataset provided by the European Centre for Medium-Range Weather Forecasts via Google Earth Engine for each study population and sampling date. In addition, nighttime Land Surface Temperature (LST) data were obtained from the MODIS MOD11A1 product. Data was extracted for the nighttime before every sample date. While LST data were collected for the night preceding each sampling event, cloud cover resulted in intermittent data gaps; consequently, air temperature was prioritized in the final models due to its continuous coverage.

### Statistical analysis

To analyse the effects of the landscape (proportion of impervious, green and water areas in buffer size of 150, 600 and 1000 m), air temperature, LST, position of the flower on the inflorescence, corolla length and diameter on nectar volume and concentration, we used generalized linear mixed models (GLMMs) implemented in the glmmTMB package. To account for potential non-linear microclimatic effects, air temperature was modeled using second-order polynomials. To prevent multicollinearity among landscape variables measured at different scales (150, 600 and 1000 m), each buffer size was analysed in separate models. Correlations among predictors were assessed using Pearson correlation coefficients and variance inflation factors (VIF). Air temperature and nighttime LST were strongly correlated (Pearson’s *r* = 0.77). Therefore, air temperature was used, as it provided complete data coverage for all sampling days. Inflorescence nested within population (population ID) and sampling date were included as random effects.

Nectar volume was analysed using GLMMs implemented in the glmmTMB package [10] with a Tweedie distribution and log link, as these variables were continuous, right-skewed and included many small values [24]. The Tweedie compound Poisson-gamma distribution was preferred over a standard Gamma distribution because 23% of sampled flowers contained no measurable nectar (0 µl). Tweedie models handle continuous, right-skewed data with a point mass at zero without requiring arbitrary data transformations. Nectar concentration, expressed as the interval between 0 and 1, was modelled using a beta distribution.

Model selection was performed using an information-theoretic approach based on Akaike’s Information Criterion corrected for small sample sizes (AICc) using the MuMIn::dredge() function [6]. Model diagnostics were assessed using simulated residuals from the DHARMa package to test for dispersion and zero inflation [21]. Model predictions were visualized as marginal responses, i.e., predicted values assuming all other predictors at a constant (mean) level using the ggeffects::ggpredict() function [34]. All statistical analyses and visualizations were performed in R version 4.1.1 [43].

## Results

**Fig. 2:**
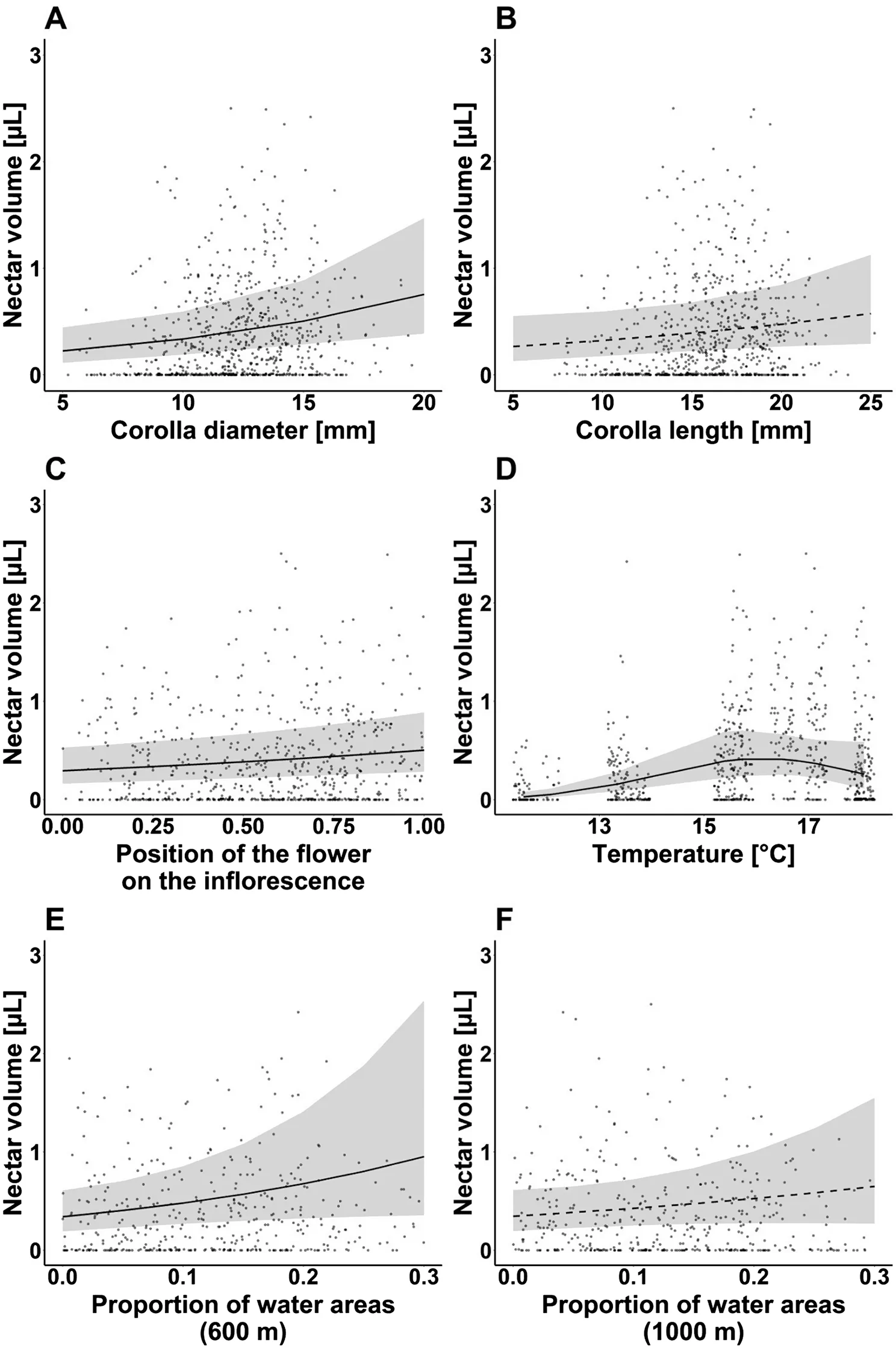
Effects of floral traits, landscape composition, and temperature on nectar volume in *Echium vulgare*. The plots show marginal effects of corolla diameter, corolla length, flower position, air temperature, and proportion of water bodies (600 and 1000 m radii). Solid lines indicate statistically significant relationships (*p* < 0.05), whereas dashed lines represent non-significant associations (*p* > 0.05). Shaded areas represent 95% confidence intervals. Full statistical parameter estimates (β, SE, z-values, and p-values) are detailed in Table 1

The GLMM analysis revealed that nectar volume was influenced by both floral morphology and environmental factors, with local microclimate and surrounding water bodies exhibiting the strongest positive associations (Table 1). Among floral traits, corolla diameter was positively related to nectar volume, whereas corolla length showed a weak positive trend (Fig. 2A, B; Table 1). Flower position along the inflorescence had a marked positive effect, with top-positioned flowers producing higher nectar volume than basal ones (Fig. 2C; Table 1).

Regarding environmental predictors, morning air temperature showed a highly significant non-linear, hump-shaped relationship with nectar volume, with an optimal microclimatic range for nectar production (Fig. 2D; Table 1). Among landscape metrics, higher coverage of blue infrastructure within 600 m and 1000 m increased nectar volume (Fig. 2E, F; Table 1). In contrast, the proportion of impervious surfaces and green areas across all analyzed spatial scales (150, 600, and 1000 m) had no impact on nectar volume (Table 1).

**Table 1:** Results of generalized linear mixed models (GLMMs) examining the effects of floral morphology, flower position, temperature, and landscape variables on nectar volume

Nectar volume	Water 600 m				Water 1000 m			
*Predictors*	*Estimate*	*SE*	*z*	*p*	*Estimate*	*SE*	*z*	*p*
Intercept	-35.685	10.444	-3.417	**0.001**	-34.705	9.675	-3.587	**< 0.001**
Corolla length	0.038	0.022	1.742	0.081	0.040	0.022	1.827	0.068
Corolla diameter	0.081	0.027	3.036	**0.002**	0.081	0.027	3.030	**0.002**
Flower position	0.534	0.169	3.169	**0.002**	0.538	0.169	3.187	**0.001**
Air temperature (linear)	4.063	1.426	2.850	**0.004**	3.911	1.320	2.963	**0.003**
Air temperature (quadratic)	-0.126	0.048	-2.633	**0.008**	-0.120	0.044	-2.718	**0.007**
Water proportion (600 m)	3.409	1.655	2.060	**0.039**				
Water proportion (1000 m)					2.085	1.595	1.307	0.191
**Random Effects**								
σ^2^	0.63				0.63			
τ_00_	0.21 _inflorescence: population.id_				0.21 _inflorescence: population.id_			
	0.16 _population.id_				0.20 _population.id_			
	0.27 _date_				0.22 _date_			
N	5 _inflorescence_				5 _inflorescence_			
	24 _population.id_				24 _population.id_			
	11 _date_				11 _date_			
Observations	733				733			

Models were fitted using a Tweedie distribution with a log link. Population identity nested within inflorescence and sampling date were included as random effects. Separate models were constructed for the proportion of water bodies at the 600 m and 1000 m spatial scales. Significant effects are indicated in bold (*p* < 0.05)

**Fig. 3:**
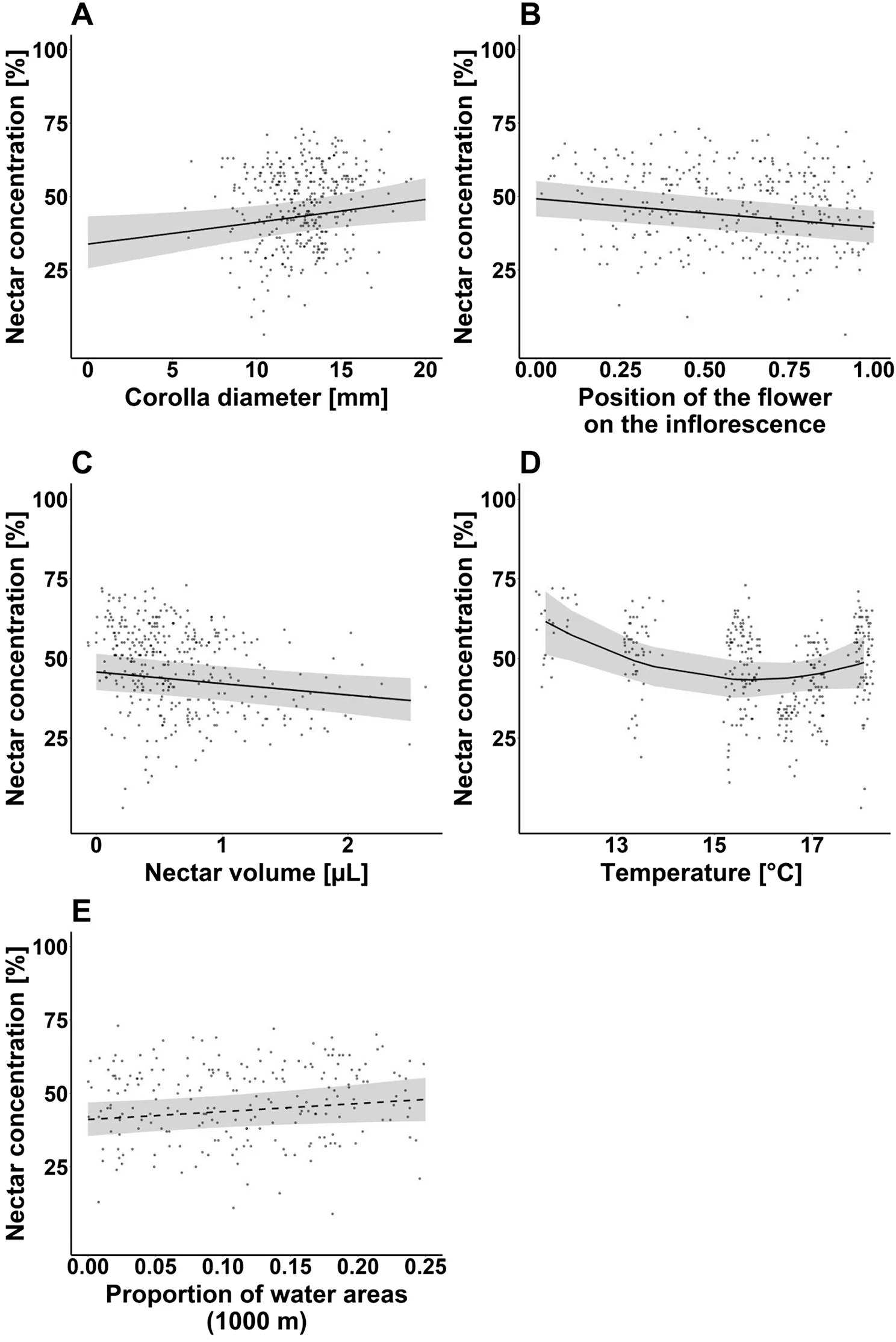
Effects of floral traits, landscape composition, and temperature on nectar concentration in *Echium vulgare*. The plots show marginal effects of corolla diameter, flower position, air temperature, nectar volume and proportion of water bodies within a 1000 m buffer. Solid lines indicate statistically significant relationships (*p* < 0.05), whereas dashed lines represent non-significant associations (*p* > 0.05). Shaded areas represent 95% confidence intervals. Full statistical parameter estimates (β, SE, z-values, and p-values) are detailed in Table 2

Nectar sugar concentration was influenced by both floral traits and local environmental conditions (Table 2). Among floral and reward traits, corolla diameter was positively associated with sugar concentration (Fig. 3A; Table 2). In contrast, nectar volume and relative flower position within the inflorescence both were negatively associated with concentration (Fig. 3B, C; Table 2).

Regarding microclimatic predictors, morning air temperature exhibited a significant non-linear, U-shaped effect on nectar concentration (Fig. 3D; Table 2). Among landscape features, the proportion of water bodies at the 1000 m scale showed a weak positive trend (Fig. 3E; Table 2). No effects were detected for the proportion of water bodies at 150 and 600 m, nor for impervious or green areas at any of the investigated spatial scales (Table 2).

**Table 2:** Results of generalized linear mixed models (GLMMs) examining the effects of nectar volume, floral morphology, temperature, and landscape variables on nectar sugar concentration

Nectar concentration	Water 1000 m			
*Predictors*	*Estimate*	*SE*	*z*	*p*
Intercept	9.934	4.544	2.186	**0.029**
Nectar volume	-0.148	0.056	-2.659	**0.008**
Corolla diameter	0.032	0.013	2.360	**0.018**
Flower position	-0.394	0.091	-4.325	**< 0.001**
Air temperature (linear)	-1.314	0.619	-2.123	**0.034**
Air temperature (quadratic)	0.042	0.021	2.008	**0.045**
Water proportion (1000 m)	1.105	0.599	1.843	0.065
**Random Effects**				
σ^2^	0.04			
τ_00 inflorescence: population.id_	0.06			
τ_00 population.id_	0.01			
τ_00 date_	0.05			
N _inflorescence_	5			
N _population.id_	24			
N _date_	11			
Observations	356			

Models were fitted using a beta distribution. Population identity nested within inflorescence and sampling date were included as random effects. Significant effects are indicated in bold (*p* < 0.05)

## Discussion

In this study, we examined variation in nectar volume and sugar concentration of *E. vulgare* across urban populations in relation to local temperature, landscape composition, and floral traits. Our results show that nectar volume and concentration were not associated with broad land-cover metrics, such as the proportion of impervious or green areas. Instead, variation in nectar volume and concentration was better explained by local thermal conditions, flower-level traits, and one component of landscape composition — the proportion of surrounding water bodies. This pattern suggests that the relevance of landscape composition depended on the specific land-cover category considered, with water-body cover showing clearer associations with nectar traits than impervious-surface or green-area cover, particularly in resilient synanthropic species. This emphasis on conditions at the plant’s growing site is expected for sessile organisms, which experience environmental variation primarily where they are established. This contrasts with mobile organisms, such as pollinators, whose responses may integrate conditions across larger areas traversed during foraging [30].

The proportion of water bodies, although quantified as a landscape-scale variable, may also covary with unmeasured local microclimatic conditions experienced by plants. It may represent more than a simple land-cover category, potentially acting as an indirect indicator of local humidity and microclimatic conditions experienced by plants. The positive association between water-body cover and nectar traits suggests that this component of landscape composition may be relevant to floral reward production [22]. Water bodies may influence surrounding microclimatic conditions through evaporative cooling [55] but we did not measure humidity, vapor pressure deficit, soil moisture, or plant physiological status. Therefore, the mechanism underlying the observed association remains unresolved.

The hump-shaped relationship between air temperature and nectar volume, together with the U-shaped response of sugar concentration, suggests that warmer urban conditions may alter the balance between nectar quantity and quality in a non-linear manner. Rather than indicating a fixed resistance of nectar traits to temperature variation, this pattern points to a contrasting nonlinear associations of temperature with nectar volume and sugar concentration. Flowers contained more nectar up to an optimal temperature before volume declined, whereas concentration initially decreased and subsequently increased, although the physiological processes underlying this pattern remain unresolved. While extreme temperatures often reduce nectar volume due to physiological stress or resource limitations [18, 49], the moderate warming observed in our study may be associated with a shift in nectar traits, although the physiological basis of this pattern remains unclear [48]. Although larger nectar volumes may influence pollinator foraging decisions [36], the ecological significance of this pattern cannot be fully assessed without estimating pollinator visitation and efficiency.

Beyond the associations with environmental variables, nectar traits also covaried with floral morphology. In our study, larger corolla diameter and length were associated with increased nectar volume, and corolla diameter was positively associated with nectar concentration. These results suggest that larger flowers, particularly those with a greater corolla diameter, were associated with greater nectar rewards per flower. This correlation may partly reflect allometric relationships between flower size and nectar-producing structures, as the ring-shaped nectary of *E. vulgare* is located at the base of the corolla tube. In line with this, larger corollas are generally associated with higher nectar volumes [13]. For instance, flowers with longer corollas tend to produce more nectar [25, 50].

Flower position within the inflorescence significantly influenced nectar traits, with upper flowers producing larger but more dilute nectar rewards. Although many species exhibit declining nectar production toward distal flowers, the contrasting pattern observed here suggests that positional reward gradients are species-specific and may reflect differences in floral resource allocation strategies [7, 54].

The observed variation in nectar traits, driven by the interplay between floral morphology and local environmental conditions, may have important implications for plant–pollinator interactions. In our study, the increase in nectar volume with temperature, coupled with a tendency toward lower sugar concentration, suggests a shift in floral rewards toward higher quantity but lower energetic quality. Such changes may influence pollinator foraging behaviour, as both nectar volume and sugar concentration determine reward value and handling efficiency [37]. Moreover, the positive relationship between corolla size and nectar rewards indicates that floral morphology may provide information about potential rewards amidst the sensory noise of the city.

The absence of associations with impervious-surface and green-area cover further emphasizes that these commonly used urbanization metrics did not capture the observed variation in nectar traits, whereas water-body cover showed clearer relationships. For a sessile organism, such a pattern is expected, as nectar production should be most associated with environmental conditions experienced at the site where the plant is rooted, whereas broader landscape structure may influence nectar traits only indirectly. Our results align with the findings of Biella et al., [8], suggesting that components of the urban landscape may vary in their relevance to nectar traits [8, 51].

The observed patterns indicate variation in nectar traits across urban conditions, but they do not allow us to determine whether this variation reflects phenotypic plasticity. Nectar traits did not remain fixed across urban gradients; instead we observed variable nectar volume and concentration in relation to local temperature, water-body cover, and flower-level traits. Our results indicate that this species may sustain floral reward function under heterogeneous urban conditions. This capacity is ecologically plausible given the species’ ruderal life history. As a plant commonly occurring in disturbed habitats, *E. vulgare* is regularly exposed to resource limitation, thermal variability, and fluctuating water availability — conditions that closely resemble many urban stressors [33]. The selective pressures in these habitats — high variability in temperature and water availability — closely mirror the stressors of urban ecosystems. Species adapted to such habitats often exhibit high physiological plasticity, allowing them to maintain key functions under changing conditions [9].

While our study provides robust insights across a large sample size, certain limitations should be considered. We did not directly measure micro-environmental relative humidity or physiological indicators of plant water status. Consequently, the observed associations between blue infrastructure and nectar traits remain indirect proxies for local microclimate and water balance. Additionally, we evaluated nectar availability but did not track actual pollinator visits or energetic intake, which limits direct conclusions about pollinator fitness in these sites.

## Conclusion

Overall, our results show that nectar traits in *E. vulgare* are associated with air temperature, floral traits, and water-body cover, whereas impervious-surface and green-area cover showed no clear relationships. In particular, air temperature, water-body cover, and floral morphology play a key role in structuring nectar volume and concentration. These patterns indicate that associations with nectar traits differed among environmental predictors and spatial scales.

This highlights the importance of considering individual components of landscape composition, environmental conditions, and floral traits when interpreting variation in urban nectar resources.

## Data Availability

The datasets generated and analysed during the current study are available in the “Dane Badawcze UW, V1” repository, https://doi.org/10.58132/P4PBTA.
